# Lactate promotes mitochondrial biogenesis and fusion via GPR81 to enhance synaptic plasticity-related protein expression in hippocampal cells

**DOI:** 10.3389/fcell.2026.1929409

**Published:** 2026-09-29

**Authors:** Qinghui Shang, Xuepeng Bian, Shujie Lou

**Affiliations:** 1 College of Sports and Health, Shandong Sport University, Jinan, China; 2 School of Exercise and Health Sciences, Shanghai University of Sport, Shanghai, China; 3 Department of Rehabilitation, School of International Medical Technology, Shanghai Sanda University, Shanghai, China

**Keywords:** GPR81, lactate, mitochondrial biogenesis, mitochondrial fusion, synaptic plasticity

## Abstract

**Background:**

Beyond its traditional role as a glycolysis end-product, lactate is now recognized as an important signaling molecule influencing synaptic plasticity and mitochondrial activity.

**Objective:**

We examined whether lactate, acting through its receptor GPR81, improves synaptic plasticity-related protein expression by augmenting mitochondrial biogenesis and fusion.

**Methods:**

Primary hippocampal cells and Neuro-2A cells were studied. Hippocampal cells received L-lactate or the GPR81 agonist 3OBA. We measured ATP production and levels of proteins involved in mitochondrial biogenesis (PGC-1α, NRF1/2, TFAM), fusion (OPA1, MFN2), and synaptic plasticity (BDNF, PSD95, SYN). In Neuro-2A cells, pathways were blocked using the PGC-1α inhibitor SR-18292 or the fusion inhibitor MF18 after lactate/GPR81 activation.

**Results:**

L-lactate and 3OBA significantly enhance mitochondrial function, including increasing ATP production, upregulating key regulators of mitochondrial biogenesis (PGC-1α, NRF1/2, and TFAM), and promoting fusion proteins (OPA1 and MFN2) that maintain mitochondrial network integrity. These mitochondrial improvements were paralleled by elevated expression of synaptic plasticity-related protein expression (BDNF, PSD95, and SYN), suggesting functional coupling between metabolic and synaptic processes. Conversely, pharmacological inhibition of mitochondrial biogenesis (via PGC-1α inhibitor SR-18292) or fusion (using MF18) abolished lactate/GPR81-mediated benefits, resulting in reduced ATP levels, impaired mitochondrial membrane potential, and downregulation of synaptic proteins.

**Conclusion:**

Our findings reveal that lactate promotes mitochondrial biogenesis and fusion via GPR81 to enhance synaptic plasticity-related protein expression in hippocampal cells, highlighting a novel mechanism by which lactate supports neuronal energy demands and synaptic plasticity.

## Introduction

Mitochondria are central to cellular energy production via oxidative phosphorylation (OXPHOS) and are also involved in calcium buffering, redox balance, lipid metabolism, and apoptosis (Nunnari and Suomalainen, 2012). In neurons, mitochondria contribute to regulation of synaptic plasticity (Harris et al., 2012; Mattson et al., 2008), with somatically generated mitochondria undergoing anterograde transport to axonal compartments where they locally supply ATP. The quality control of these axonal mitochondria is maintained through two key mechanisms: enhanced mitochondrial biogenesis and selective mitochondrial fusion between healthy and damaged organelles (Zheng et al., 2019). This dynamic quality control system underscores the importance of mitochondrial integrity for sustaining normal synaptic function.

Growing evidence indicates that deficits in mitochondrial biogenesis or fusion machinery are strongly associated with synaptic dysfunction. These two interconnected processes - biogenesis and fusion - work in concert to regulate both mitochondrial quantity and subcellular distribution, thereby meeting the elevated energy demands required for synaptic plasticity. Therefore, promoting mitochondrial biogenesis and fusion is crucial for mitochondrial function. PGC-1α is a major driver of mitochondrial biogenesis; its knockdown inhibits BDNF to promote dendritic spine formation (Cheng et al., 2012), while its overexpression improves the mitochondrial respiration, BDNF expression, and cognitive performance (Han et al., 2020). Similarly, mitochondrial fusion is equally important for mitochondrial transport and function (Misko et al., 2010), with direct implications for synaptic plasticity (Li et al., 2004). Collectively, these findings establish that coordinated mitochondrial biogenesis and fusion optimize neuronal energy metabolism, ultimately supporting enhanced neuronal function.

Once regarded merely as a glycolytic waste product, lactate is now known to facilitate synaptic plasticity (Herrera-López et al., 2020; Margineanu et al., 2018; Yang et al., 2014). Notably, recent studies reveal its additional role in enhancing mitochondrial function. In rat hippocampi, lactate treatment can upregulate expression of mitochondrial biogenesis regulators and antioxidant defense systems (Akter et al., 2023a). Importantly, cortical lactate deficiency has been linked to impaired memory formation through suppressed mitochondrial biogenesis—a phenotype reversible by L-lactate administration through PGC-1α induction and mtDNA restoration (Akter et al., 2023b). These observations suggest lactate may help neurons produce more ATP to fuel neuronal activity and plasticity.

The lactate receptor GPR81 is broadly expressed throughout the brain parenchyma, with particularly high density in hippocampal and neocortical pyramidal neurons where it responds to physiological concentrations of lactate (Lauritzen et al., 2014). As lactate serves as the sole known endogenous ligand for GPR81 (Liu et al., 2009), this receptor provides a molecular mechanism for lactate’s signaling functions in the brain. Through GPR81 activation, lactate orchestrates diverse neurobiological processes including: modulation of neuronal excitability (Briquet et al., 2022; Skwarzynska et al., 2023), regulation of angiogenesis (Morland et al., 2017; Chaudhari et al., 2022) and neurogenesis (Lambertus et al., 2020; Kennedy et al., 2022), and control of neuroinflammatory responses (Kennedy et al., 2022). Importantly, emerging evidence suggests GPR81’s role in mitochondrial regulation, including promoting mitochondrial biogenesis in glioblastoma cells (Longhitano et al., 2022a), enhancing ATP production in retinal tissue (Vohra et al., 2022), and improving mitochondrial respiratory function in Müller cells (Vohra et al., 2019). While our previous work established the role of the GPR81-ERK1/2 pathway in lactate-mediated hippocampal mitochondrial function (Shang et al., 2023), the specific downstream mitochondrial processes targeted by this pathway, and whether these processes directly underpin the beneficial effects on synaptic plasticity, remain unexplored. Addressing this knowledge gap is essential for understanding lactate’s neuroprotective mechanisms.

Based on the above, we proposed that lactate enhances mitochondrial function through GPR81 activation, thereby promoting molecular events associated with synaptic plasticity. To test this hypothesis, we employed a dual experimental approach. We tested this using primary hippocampal cells treated with L-lactate or a GPR81 agonist to assess mitochondrial and synaptic markers, and Neuro-2A cells in which we inhibited PGC-1α or mitochondrial fusion to see if these disruptions block lactate-induced plasticity gains.

## Methods

### Primary hippocampal cell culture and treatment

Primary hippocampal cells were isolated from C57BL/6 mice of both sexes within 24 h after birth (P0–P1) (sourced from Jiesijie Laboratory Animal company, Certificate SCXK 2018–0014, Shanghai, China). Cells were cultured for 6 days *in vitro* (DIV 6) prior to treatment. For neonatal mice within 24 h of birth, sex determination was not performed as sexual dimorphism is not morphologically distinguishable at this stage without genotyping. Cells were plated at a density of 1 × 10^6^ cells per well in six-well plates and maintained in Neurobasal medium (Gibco, 10888022) supplemented with 2% B27 (Gibco, 17504044) and 1% Penicillin-Streptomycin Solution (Beyotime, C0222). Cells were incubated at 37 °C in a humidified atmosphere of 5% CO_2_. On day 6, cells received 15 mM L-lactate (Sigma, L7022) for 6 h or 0.5 mM GPR81 agonist 3OBA (Selleck, S5400) for 3 h. L-lactate was dissolved in Neurobasal medium and the 3OBA stock solution was prepared in DMSO and diluted in Neurobasal medium immediately before use. To control for potential solvent effects, equivalent volumes of DMSO were added to both the untreated control and L-lactate treatment groups.

### Neuro-2A cell culture and treatment

Neuro-2A cells were employed for the pharmacological inhibitor experiments as a complementary mechanistic screening platform. This cell line was chosen because it allows for efficient and reproducible pharmacological manipulations with SR-18292 and MF18, which are resource-intensive and technically challenging to perform in primary hippocampal cells. Additionally, Neuro-2A cells provide a more homogeneous cell population for dissecting specific signaling pathways. However, we acknowledge that Neuro-2A cells are a proliferative neuroblastoma line and do not fully recapitulate the physiology of post-mitotic neurons; therefore, our core findings were first established in primary hippocampal cells, and the Neuro-2A experiments serve as complementary mechanistic support. Neuro-2A cells were maintained in MEM (Invitrogen, 11090–081) containing 10% FBS (17504044, Gibco), 1% GlutaMax (Invitrogen, 35050061), 1% sodium pyruvate (Invitrogen, 11360070), 1% NEAA (Invitrogen, 11140050), and 1% penicillin-streptomycin solution (Beyotime, C0222) at 37 °C in a humidified atmosphere of 5% CO_2_. Neuro-2A cells were treated with L-lactate for 6 h or the GPR81 agonist 3OBA for 3 h. To determine the optimal concentrations for subsequent experiments, Neuro-2A cells were treated with increasing concentrations of Sodium L-lactate (5, 10, 15, and 20 mM) or the GPR81 agonist 3OBA (0.5 and 1 mM) for their respective durations. Based on dose-dependent upregulation of GPR81 protein expression (Supplementary Figure S1), 15 mM L-lactate and 0.5 mM 3OBA were selected for all mechanistic studies in both Neuro-2A cells and primary hippocampal cells. PGC-1α inhibitor SR-18292 (Selleck, S8528) or mitochondrial fusion inhibitor MF18 (Med Chem Express, HY-150031) was applied 30 min before L-lactate/3OBA. In all inhibitor experiments, cells treated with equivalent volumes of DMSO (matching the highest concentration used in inhibitor treatments) served as vehicle controls.

### mPTP opening detection

Mitochondrial permeability transition pore (mPTP) opening was assessed using a commercial assay kit (Beyotime, C2009S) according to the manufacturer’s protocol. The cells were washed twice with PBS and incubated with Calcein AM staining solution and fluorescence quenching solution per well for 30 min at 37 °C. Then, the cells were incubated with MEM for 30 min at 37 °C. Then, the cells were washed twice with PBS and 2 mL of assay buffer was added. Images were acquired using a Nikon ECLIPSE Ti2 fluorescence microscope (Nikon Corporation, Japan) with a ×40 objective lens. For quantification of fluorescence, the fluorescence signal within neurites was measured using ImageJ software. Rectangular regions of interest (ROIs) were manually drawn along the neurites for each cell, and the mean fluorescence intensity per ROI was calculated. The soma was excluded from all quantitative analyses. Because the LA and 3OBA series represent two independent experimental arms, the LA group and the 3OBA group served as the respective calibrators for their own series (each set to 1.0).

### JC-1 staining for mitochondrial membrane potential (MMP) assessment

Following the manufacturer’s instructions, mitochondrial membrane potential was evaluated with JC-1 MMP assay kit (Beyotime, C2003S). After a PBS wash, cells were treated with JC-1 working solution for 20 min at 37 °C, followed by two washes with JC-1 buffer and addition of MEM. Images (red and green channels) were acquired using a Nikon ECLIPSE Ti2 fluorescence microscope (Nikon Corporation, Japan) with a ×10 objective lens, and MMP was calculated based on the red/green fluorescence intensity ratio. Because the LA and 3OBA series represent two independent experimental arms, the LA group and the 3OBA group served as the respective calibrators for their own series (each set to 1.0).

### ATP detection

ATP content was assessed with an enhanced ATP assay kit (Beyotime, S0027). Primary hippocampal cells and Neuro-2A cells were processed separately. After lysis in ATP lysis buffer on ice, samples were centrifuged at 12,000 × g for 5 min at 4 °C. The collected supernatants were then subjected to analysis. For the measurement, 100 µL of ATP working solution and 20 µL of each supernatant were combined in a 96-well plate and mixed promptly. A luminometer was used to record ATP-dependent luminescence. ATP levels were normalized to protein concentration for each sample and then expressed relative to the designated calibrator for each experimental series (CON for primary neuron experiments; LA or 3OBA for their respective series in Neuro-2A experiments).

### RNA extraction and reverse transcription–quantitative PCR (RT-qPCR)

RNA extraction and cDNA synthesis were performed sequentially. First, total RNA was extracted from cells and tissues with TRIzol reagent (Takara, 9109). Reverse transcription was then conducted using the PrimeScript™ RT reagent Kit (Takara, RR047A) per the protocol. Finally, quantitative PCR was executed with TB Green® Premix Ex Taq™ II (Takara, RR820A) on a QuantStudio 3 Real-Time PCR System (Thermo Fisher Scientific, United States of America). The thermal cycling protocol was as follows: initial denaturation at 95 °C for 30 s, followed by 40 cycles of 95 °C for 5 s and 60 °C for 34 s. A melt curve analysis was performed to verify amplification specificity: 95 °C for 15 s, 60 °C for 60 s, and 95 °C for 15 s. All reactions were run in triplicate, and relative gene expression was calculated using the 2^^–ΔΔCt^ method. For primary hippocampal neuron experiments, the CON group served as the calibrator. For Neuro-2A cell experiments, the LA group and the 3OBA group served as the respective calibrators for their own series. The calibrator group was set to 1.0 for all genes. Corresponding primer sequences are detailed in Table 1.

**TABLE 1:** Primer sequences for RT-qPCR analysis.

Genes	Primer	Sequence (5′–3′)
NDUFS8	Forward	AGT​GGC​GGC​AAC​GTA​CAA​G
	Reverse	TCG​AAA​GAG​GTA​ACT​TAG​GGT​CA
SDHb	Forward	AAT​TTG​CCA​TTT​ACC​GAT​GGG​A
	Reverse	AGC​ATC​CAA​CAC​CAT​AGG​TCC
Uqcrc1	Forward	ACG​CAA​GTG​CTA​CTT​CGC​A
	Reverse	CAG​CGT​CAA​TCC​ACA​CTC​CC
COX5b	Forward	TCT​AGT​CCC​GTC​CAT​CAG​CAA​C
	Reverse	GCA​GCC​AAA​ACC​AGA​TGA​CAG​T
Atp5a1	Forward	TCT​CCA​TGC​CTC​TAA​CAC​TCG
	Reverse	CCA​GGT​CAA​CAG​ACG​TGT​CAG
PGC-1α	Forward	TTC​ATC​TGA​GTA​TGG​AGT​CGC​T
	Reverse	GGG​GGT​GAA​ACC​ACT​TTT​GTA​A
NRF1	Forward	GTG​GGA​CAG​CAA​GCG​ATT​GTA
	Reverse	TTG​TAC​TTT​CGC​ACC​ACA​TTC​T
TFAM	Forward	CAA​AGG​ATG​ATT​CGG​CTC​AGG
	Reverse	TCG​ACG​GAT​GAG​ATC​ACT​TCG
OPA1	Forward	TGG​AAA​ATG​GTT​CGA​GAG​TCA​G
	Reverse	CAT​TCC​GTC​TCT​AGG​TTA​AAG​CG
MFN2	Forward	TGA​CCT​GAA​TTG​TGA​CAA​GCT​G
	Reverse	AGA​CTG​ACT​GCC​GTA​TCT​GGT
Arc	Forward	AAG​TGC​CGA​GCT​GAG​ATG​C
	Reverse	CGA​CCT​GTG​CAA​CCC​TTT​C
Fos	Forward	CGG​GTT​TCA​ACG​CCG​ACT​A
	Reverse	TTG​GCA​CTA​GAG​ACG​GAC​AGA
Egr1	Forward	TCG​GCT​CCT​TTC​CTC​ACT​CA
	Reverse	CTC​ATA​GGG​TTG​TTC​GCT​CGG
Bdnf	Forward	TCA​TAC​TTC​GGT​TGC​ATG​AAG​G
	Reverse	AGA​CCT​CTC​GAA​CCT​GCC​C
Syp	Forward	CAG​TTC​CGG​GTG​GTC​AAG​G
	Reverse	ACT​CTC​CGT​CTT​GTT​GGC​AC
GAPDH	Forward	AGG​TCG​GTG​TGA​ACG​GAT​TTG
	Reverse	TGT​AGA​CCA​TGT​AGT​TGA​GGT​CA

### Western blot analysis

Western blot analysis was conducted. Protein extraction from cells was performed with ice-cold RIPA lysis buffer (Beyotime, P0013B) containing 1% PMSF (Beyotime, ST506), followed by centrifugation at 14,000 × g for 5 min at 4 °C. The protein concentration of the supernatant was quantified using the BCA method (kit P0010, Beyotime). Prior to loading, samples were heated at 95 °C for 10 min in loading buffer (Beyotime, P0015L). A total of 20 µg protein was separated on 10% polyacrylamide gels (Epizyme, PG112/PG113) and electrophoretically transferred to PVDF membranes. Following transfer, membranes were blocked and incubated overnight at 4 °C with the indicated primary antibodies: GPR81 (1:400, Sigma, SAB1300790), p-ERK1/2 (1:1000, CST, 4370), ERK1/2 (1:1000, CST, 4695), BDNF (1:1000, Proteintech, 28205-1-AP), SYN (1:1000, CST, 4329), PSD95 (1:1000, CST, 3450), PGC-1α (1:1000, Novus, NBP1-04676SS), NRF1 (1:1000, CST, 46743), NRF2 (1:1000, CST, 12721), TFAM (1:2000, Abcam, ab131607), OPA1 (1:1000, CST, 8047), MFN1 (1:2000, Proteintech, 13798-1-AP), MFN2 (1:1000, CST, 9482), and β-Tubulin (1:2000, Proteintech, 10068-1-AP). After washing, membranes were incubated with an HRP-conjugated secondary antibody (1:5000, Proteintech, SA00001-2) for 1 h at room temperature. Detection was achieved via enhanced chemiluminescence, and band intensity quantification was performed using ImageJ software. For each sample, the expression level of each target protein was normalized to β-Tubulin as the loading control. The resulting ratio (target protein/β-Tubulin) for each treatment group was then normalized to the designated calibrator for each experimental series. For primary hippocampal neuron experiments, the CON group served as the calibrator (set to 1.0). For Neuro-2A cell experiments, because the LA and 3OBA series represent two independent experimental arms, the LA group and the 3OBA group served as the respective calibrators for their own series (each set to 1.0), allowing the assessment of inhibitor effects relative to their corresponding activator control.

### Mito-Tracker Red CMXRos staining

Mitochondria were visualized by staining with Mito-Tracker Red CMXRos (Beyotime, C1035). After two washes with PBS, cells were incubated with 2 mL of the dye working solution per well for 30 min at 37 °C. Unbound dye was removed by washing twice with PBS and the medium was replaced with 2 mL of MEM prior to imaging. Red fluorescence, indicating mitochondrial localization, was observed by a Nikon ECLIPSE Ti2 fluorescence microscope (Nikon Corporation, Japan) with a ×40 objective lens. For quantification of mitochondrial distribution, the fluorescence signal within neurites was measured using ImageJ software. Rectangular regions of interest (ROIs) were manually drawn along the neurites for each cell, and the mean fluorescence intensity per ROI was calculated. The soma was excluded from all quantitative analyses. Because the LA and 3OBA series represent two independent experimental arms, the LA group and the 3OBA group served as the respective calibrators for their own series (each set to 1.0).

### Statistical analysis

In all experiments, n represents the number of independent biological replicates. For primary hippocampal neuron experiments, each biological replicate (n = 1) corresponds to a separate culture preparation derived from an individual litter of newborn C57BL/6 mice, with treatments and analyses performed on different days. For Neuro-2A cell experiments, each biological replicate (n = 1) corresponds to an independently passaged culture batch from separate experimental runs. Within each biological replicate, technical replicates (e.g., multiple wells per condition) were averaged to obtain a single value for that replicate. All data are presented as mean ± SD from at least three independent biological replicates. Data processing and analysis were performed in GraphPad Prism version 8.0. Data were analyzed using one-way ANOVA followed by Bonferroni’s *post hoc* test for multiple comparisons. For three-group experiments (primary hippocampal cells), a single one-way ANOVA was performed comparing CON, LA, and 3OBA groups. For six-group experiments (Neuro-2A cells), two independent one-way ANOVAs were performed: one comparing the three L-lactate treatment groups (LA, LA + SR, LA + MF), and the other comparing the three 3OBA treatment groups (3OBA, 3OBA + SR, 3OBA + MF). This approach was adopted because the LA and 3OBA series represent two independent experimental arms with distinct pharmacological activators. Prior to ANOVA, data normality was assessed using the Shapiro–Wilk test and homogeneity of variances was evaluated using Levene’s test; all data met the assumptions for parametric analysis (*p* > 0.05). All data are presented as mean ± SD from at least three independent biological replicates. Statistical significance was defined as *p* < 0.05.

## Results

### Optimization of L-lactate and 3OBA concentrations in Neuro-2A cells

To establish appropriate concentrations for GPR81 activation, Neuro-2A cells were treated with increasing doses of Sodium L-lactate (5, 10, 15, and 20 mM) or 3OBA (0.5 and 1 mM). Western blot analysis revealed that 15 mM L-lactate and 0.5 mM 3OBA each induced the highest GPR81 protein expression among their respective tested concentrations (Figure 1). Based on these parallel optimization results, these concentrations were used in all subsequent experiments in both Neuro-2A cells and primary hippocampal cells.

**FIGURE 1:**
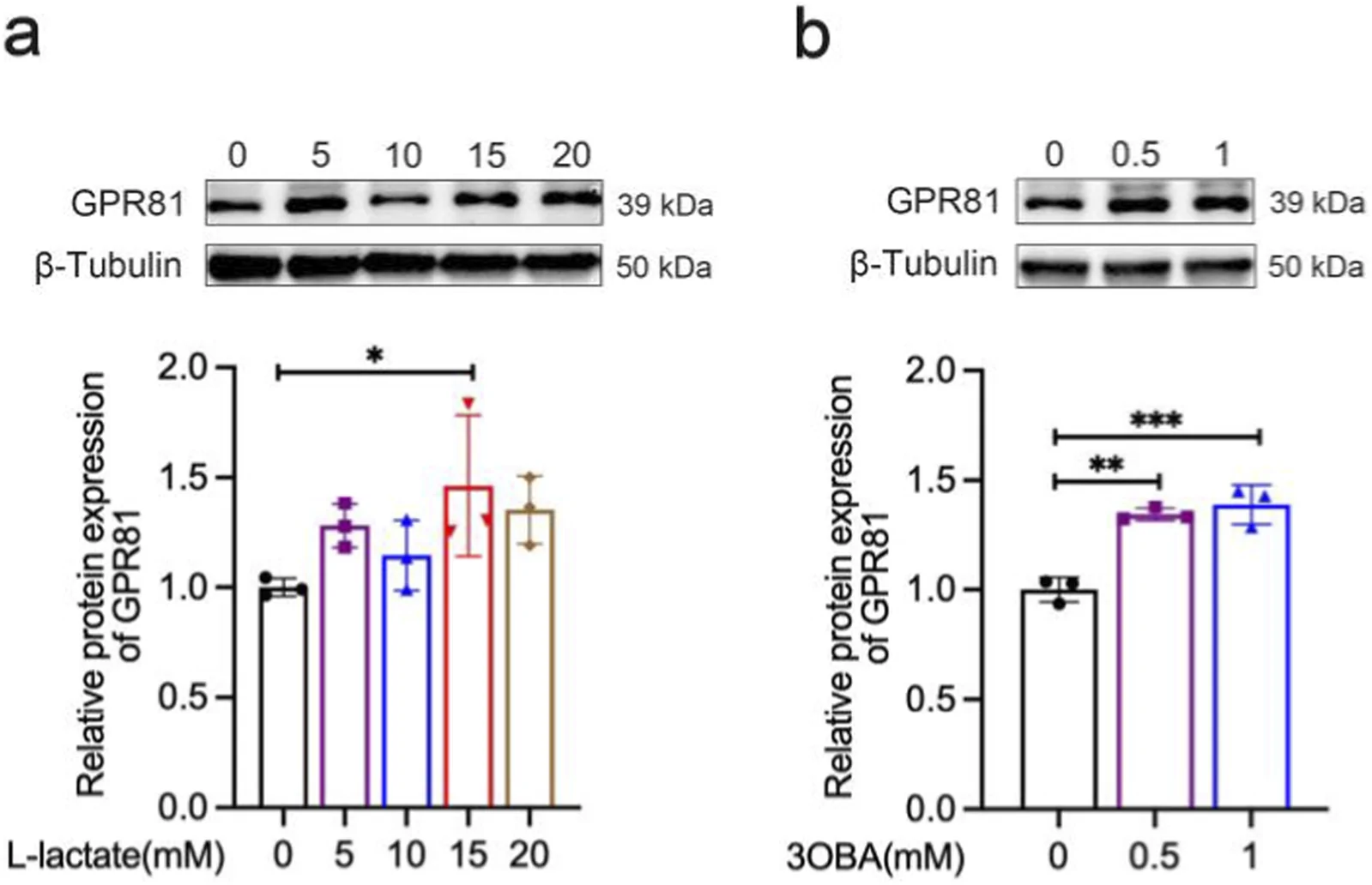
Effects of L-lactate and GPR81 agonist on GPR81 protein expression in Neuro-2A cells. **(a, b)** Exemplary immunoblotting analysis and bar graphs depict the quantified protein levels (normalized to CON) for GPR81, derived from three independent replicates. Data are expressed as mean ± SD. Statistical significance: *p < 0.05, **p < 0.01, ***p < 0.001 versus the CON group.

### L-lactate and GPR81 agonist increase ATP levels in primary hippocampal cells

Previous studies have established lactate’s role in boosting neuronal ATP generation (Jourdain et al., 2016; Jourdain et al., 2018). To investigate the involvement of lactate receptor GPR81 activation in this process, we treated the primary hippocampal cells with either L-lactate (LA) or the specific GPR81 agonist 3-chloro-5-hydroxybenzoic acid (3OBA). Western blot analysis revealed significant upregulation of GPR81 protein expression following both LA and 3OBA treatment compared to control (CON) conditions (Figure 2a). To assess whether GPR81 is functionally activated, we examined ERK1/2 phosphorylation, a well-established downstream signaling event following GPR81 activation. Both L-lactate and 3OBA treatment significantly increased ERK1/2 phosphorylation (p-ERK/t-ERK ratio) compared to the CON group (Figure 2b), confirming that GPR81 is functionally activated under our experimental conditions. Importantly, ATP quantification demonstrated markedly elevated cellular ATP levels in both treatment groups (Figure 2c). These findings provide direct evidence that both lactate and GPR81 agonist enhance energy metabolism in primary hippocampal cells.

**FIGURE 2:**
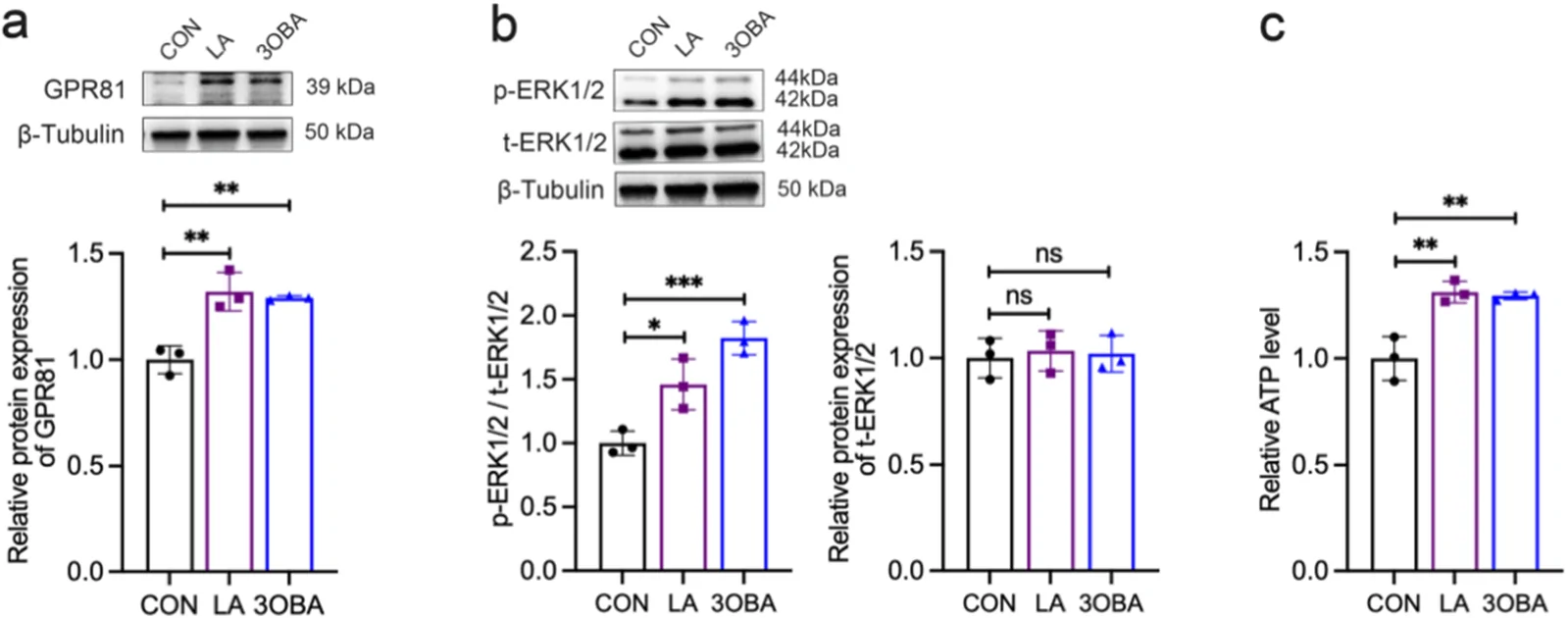
Effects of L-lactate and GPR81 agonist on ATP levels in primary hippocampal cells. **(a)** Exemplary immunoblotting analysis and bar graphs depict the quantified protein levels (normalized to CON) for GPR81, derived from three independent replicates. **(b)** Representative immunoblots of p-ERK1/2 and t-ERK1/2, and quantification normalized to CON, derived from three independent replicates. **(c)** Quantification of relative ATP levels, derived from three independent replicates. Data are expressed as mean ± SD. Statistical significance: **p < 0.01, ***p < 0.001 versus the CON group.

### L-lactate and GPR81 agonist increase the expression of proteins related to mitochondrial biogenesis and fusion in primary hippocampal cells

Quantitative analysis revealed that L-lactate (LA) treatment significantly upregulated key regulators of mitochondrial biogenesis, including PGC-1α (Figure 3b), NRF1 (Figure 3c), NRF2 (Figure 3d), TFAM (Figure 3e), compared to control (CON) conditions. Similarly, mitochondrial fusion proteins OPA1 (Figure 3f) and MFN2 (Figure 3h) were markedly elevated following LA exposure. Notably, the GPR81 agonist 3OBA produced comparable upregulation of these mitochondrial regulatory proteins, mirroring the effects of LA treatment. Regarding MFN1 (Figure 3g), 3OBA treatment significantly increased its protein expression, whereas L-lactate treatment showed no significant effect. These findings demonstrate that GPR81 activation is sufficient to enhance both mitochondria biogenesis and fusion in primary hippocampal cells. Representative immunoblots are shown in Figure 3a.

**FIGURE 3:**
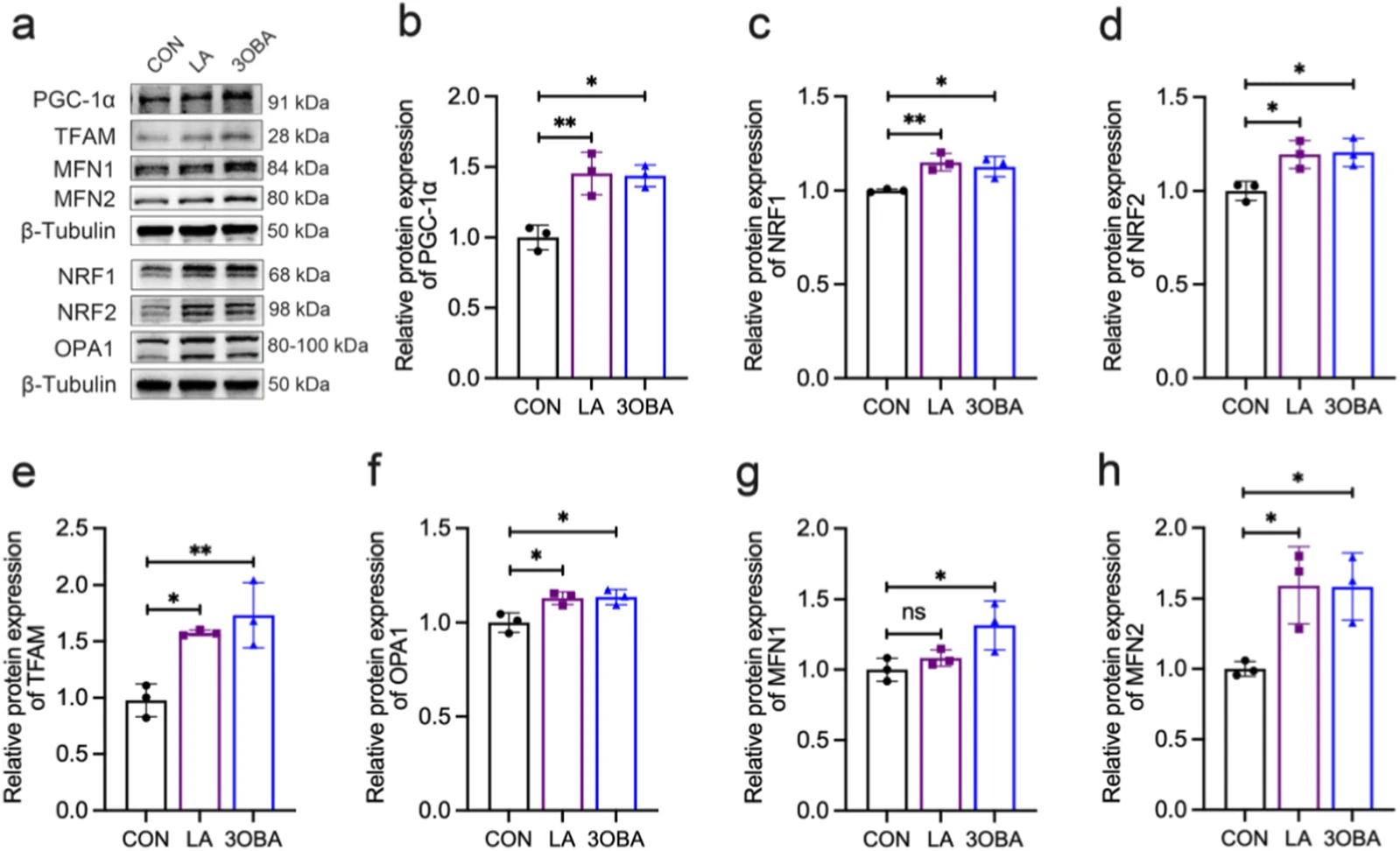
Modulation of mitochondrial biogenesis and fusion protein expression by L-lactate and GPR81 agonist in primary hippocampal cells. **(a)** Exemplary immunoblotting analysis **(b-h)** Bar graphs depict the quantified protein levels (normalized to CON) for PGC-1α, NRF1, NRF2, TFAM, OPA1, MFN1, and MFN2, derived from three independent replicates. Data are expressed as mean ± SD. Statistical significance: *p < 0.05, **p < 0.01 versus the CON group; n. s., not significant.

### L-lactate and GPR81 agonist increase the expression of proteins related to synaptic plasticity in primary hippocampal cells

As mitochondria serve as the primary energy source for neuronal activity and synaptic maintenance, we investigated whether lactate-mediated mitochondrial enhancement correlates with molecular markers of synaptic function. Immunoblot analysis revealed significantly elevated expression of synaptic plasticity-related proteins in both LA and 3OBA-treated groups compared to controls (Figure 4). These findings suggest that the observed synaptic plasticity enhancement may be mechanistically linked to lactate/GPR81-induced improvements in mitochondrial function, which provide the necessary bioenergetic support for synaptic remodeling and maintenance.

**FIGURE 4:**
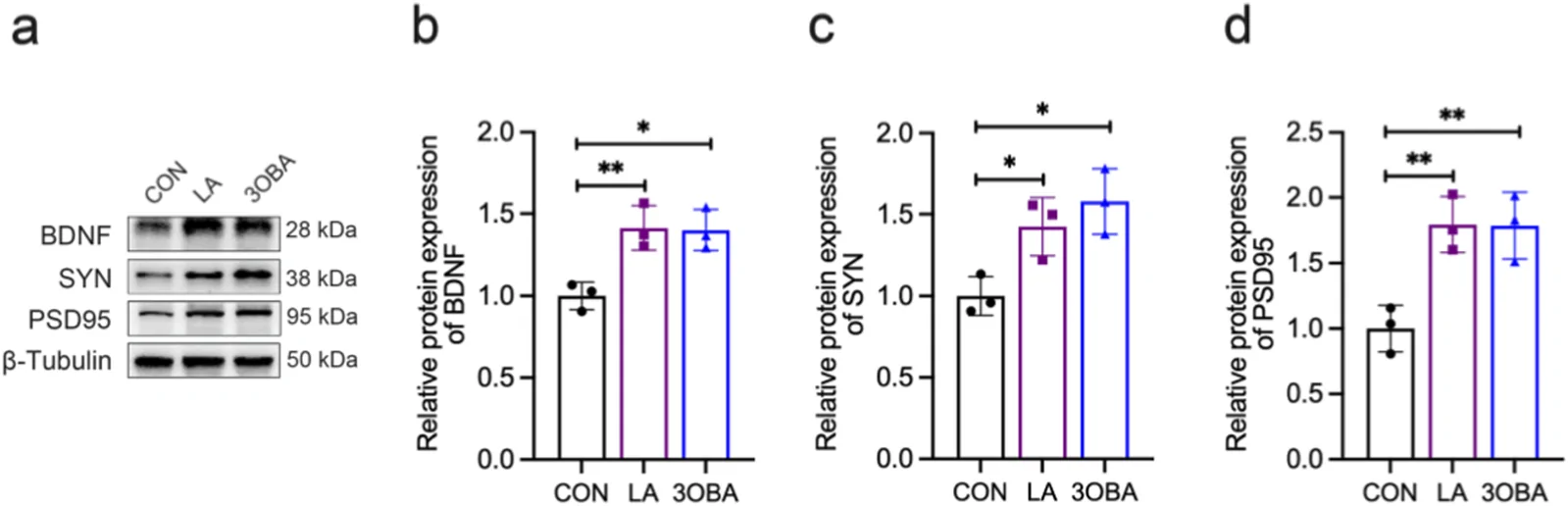
Modulation of synaptic plasticity protein expression by L-lactate and GPR81 agonist in primary hippocampal cells. **(a)** Exemplary immunoblotting analysis. **(b-d)** Bar graphs depict the quantified protein levels (normalized to CON) for BDNF, SYN, and PSD95, derived from three independent replicates. Data are expressed as mean ± SD. Statistical significance: *p < 0.05, **p < 0.01 versus the CON group.

### SR-18292 impairs L-lactate- or GPR81 agonist-induced the expression of proteins related to mitochondrial biogenesis in Neuro-2A cells

The PGC-1α inhibitor SR-18292 significantly attenuated both L-lactate (LA)- and 3OBA-induced mitochondrial biogenesis in Neuro-2A cells, as evidenced by reduced mRNA expression of *PGC-1α* (Figure 5a), *NRF1* (Figure 5b), and *TFAM* (Figure 5c), accompanied by corresponding decreases in protein levels of PGC-1α (Figure 5e), NRF1 (Figure 5f), NRF2 (Figure 5g), and TFAM (Figure 5h). Representative immunoblots are shown in Figure 5d.

**FIGURE 5:**
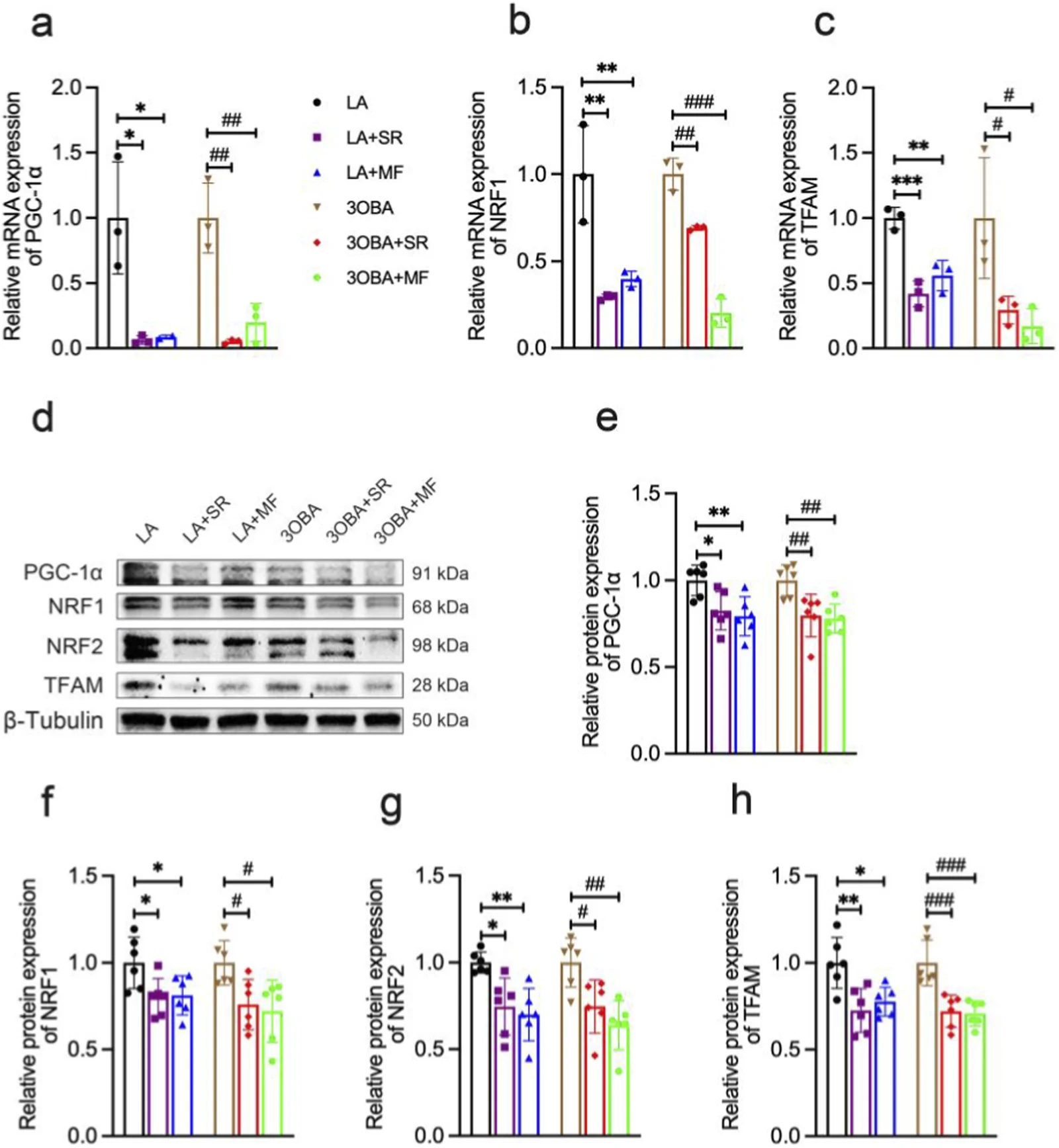
The suppressive effect of SR-18292 on genes and proteins regulating mitochondrial biogenesis. **(a-c)** Quantitative analysis (qRT-PCR) of *PGC-1α*, *NRF1*, and *TFAM* transcript levels, derived from three independent replicates. **(d)** Exemplary Western blot image. **(e-h)** Bar graphs depict the quantified protein levels for PGC-1α, NRF1, NRF2, and TFAM, derived from six independent replicates. Data are expressed as mean ± SD. Statistical significance: *p < 0.05, **p < 0.01, ***p < 0.001 versus the LA group. #*p* < 0.05, ##*p* < 0.01, ###*p* < 0.001 versus the 3OBA group.

### MF18 impairs L-lactate- or GPR81 agonist-induced the expression of proteins related to mitochondrial fusion in Neuro-2A cells

Treatment with the mitochondrial fusion inhibitor MF18 significantly suppressed both L-lactate (LA)- and 3OBA-induced mitochondrial fusion, as demonstrated by reduced OPA1 mRNA expression (Figure 6a) and significantly decreased OPA1 and MFN2 protein levels (Figure 6d,e). Notably, MFN2 mRNA showed a decreasing trend following MF18 treatment, but this change did not reach statistical significance (Figure 6b), suggesting a potential post-transcriptional regulatory mechanism for MFN2 under these conditions. Representative immunoblots are shown in Figure 6c.

**FIGURE 6:**
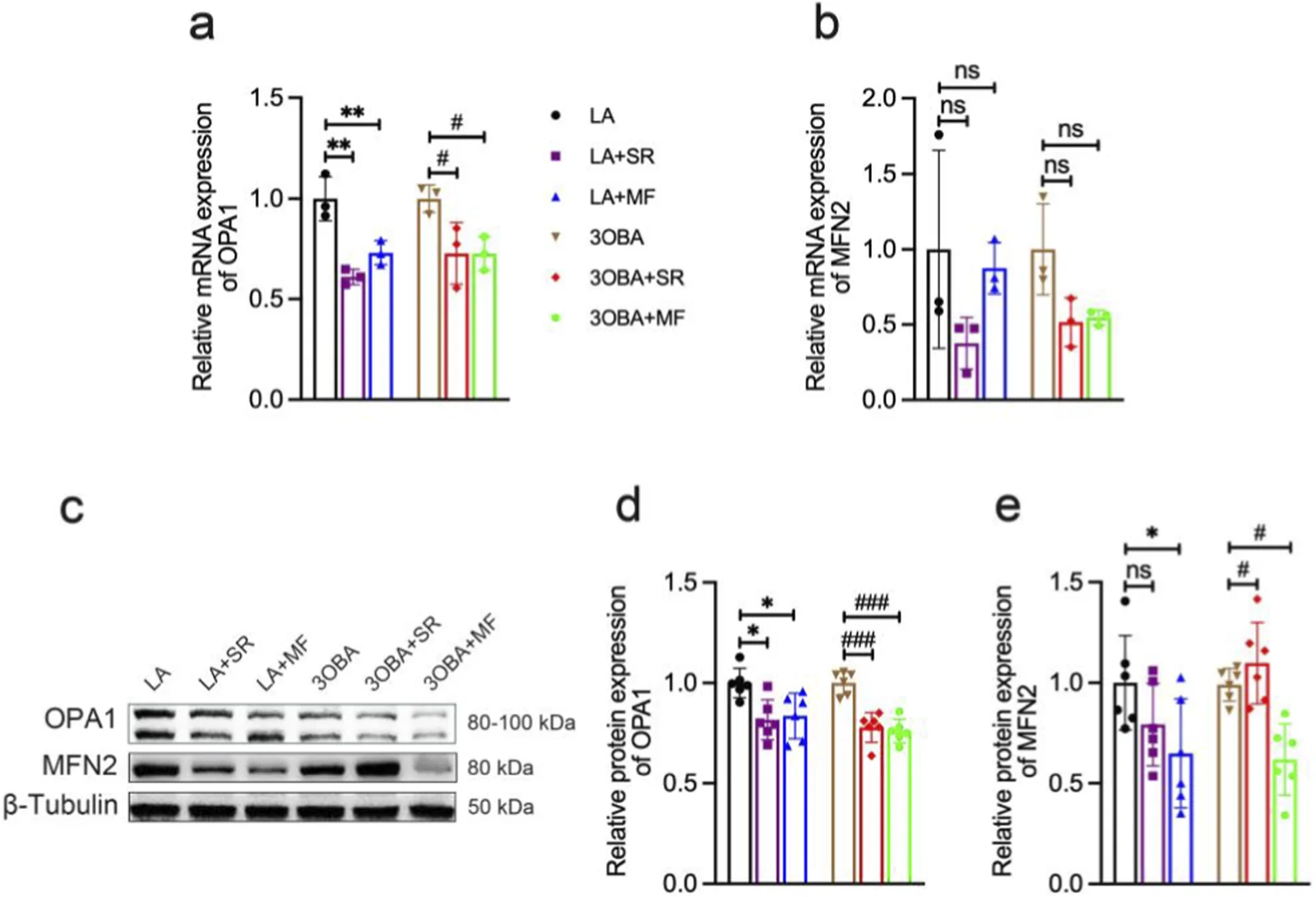
The suppressive effect of MF18 on genes and proteins regulating mitochondrial fusion **(a,b)** Quantitative analysis (qRT-PCR) of *OPA1* and *MFN2* transcript levels, derived from three independent replicates. **(c)** Exemplary Western blot image **(d,e)** Bar graphs depict the quantified protein levels for OPA1 and MFN2, derived from six independent replicates. Data are expressed as mean ± SD. Statistical significance: *p < 0.05, **p < 0.01 versus the LA group. #*p* < 0.05, ###*p* < 0.001 versus the 3OBA group. n. s., no significant difference.

### SR-18292 and MF18 facilitate the opening of the mitochondrial permeability transition pore in Neuro-2A cells

The mitochondrial permeability transition pore (mPTP) serves as a crucial indicator of mitochondrial dysfunction, where a decrease in fluorescence intensity suggests a significant elevation in its opening. mPTP analysis revealed that both SR-18292 and MF18 significantly increased mPTP opening, as evidenced by decreased fluorescence intensity in LA + SR and LA + MF groups compared to LA-treated cells (Figure 7a–c). This effect was similarly observed in 3OBA-treated cells following inhibitor exposure (Figure 7d–f), demonstrating that impairment of either mitochondrial biogenesis or fusion pathways compromises mitochondrial integrity and promotes pathological mPTP activation.

**FIGURE 7:**
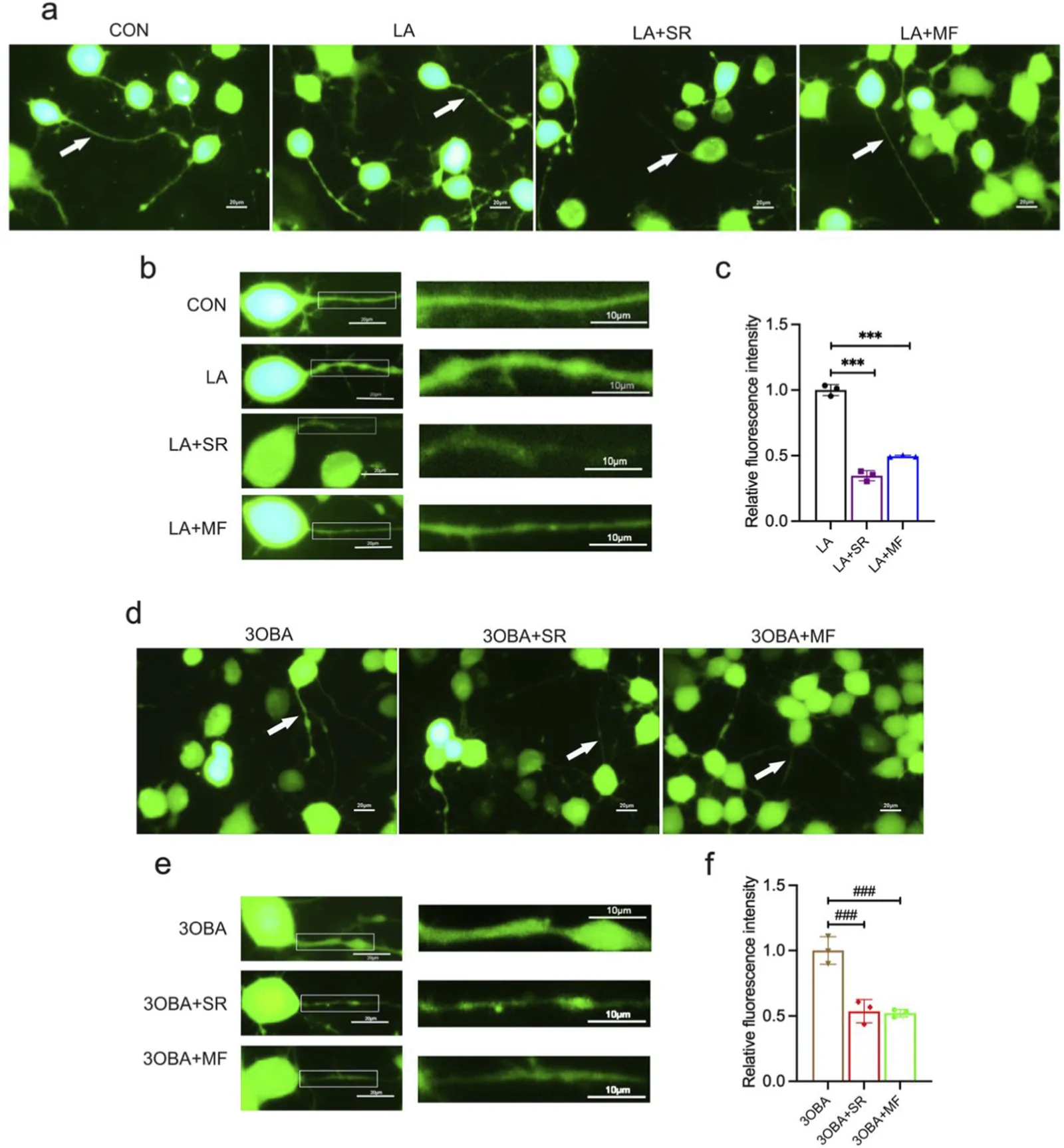
Effects of SR-18292 and MF18 on the opening of mPTP on in Neuro-2A cells. **(a-d)** Full-field overview images of monolayer cells. **(b-e)** Higher magnification cropped images with rectangular boxes indicating the neurite regions of interest (ROIs) used for fluorescence quantification; the soma was excluded from analysis. **(c-f)** Bar graphs depict the quantification of mPTP, determined by the fluorescence intensity from three replicates. Data are expressed as mean ± SD. Statistical significance: ***p < 0.001 versus the LA group. ###*p* < 0.001 versus the 3OBA group.

### SR-18292 and MF18 impair mitochondrial membrane potential in Neuro-2A cells

The opening of mPTP subsequently leads to a diminution of the mitochondrial membrane potential (MMP). Consistent with the observed mPTP opening, JC-1 staining revealed significant reductions in MMP following treatment with either SR-18292 (LA + SR) or MF18 (LA + MF) compared to LA alone (Figure 8a). This effect was similarly observed in 3OBA-treated cells (Figure 8b), demonstrating that pharmacological inhibition of either mitochondrial biogenesis (via PGC-1α suppression) or fusion machinery leads to progressive mitochondrial dysfunction characterized by membrane depolarization.

**FIGURE 8:**
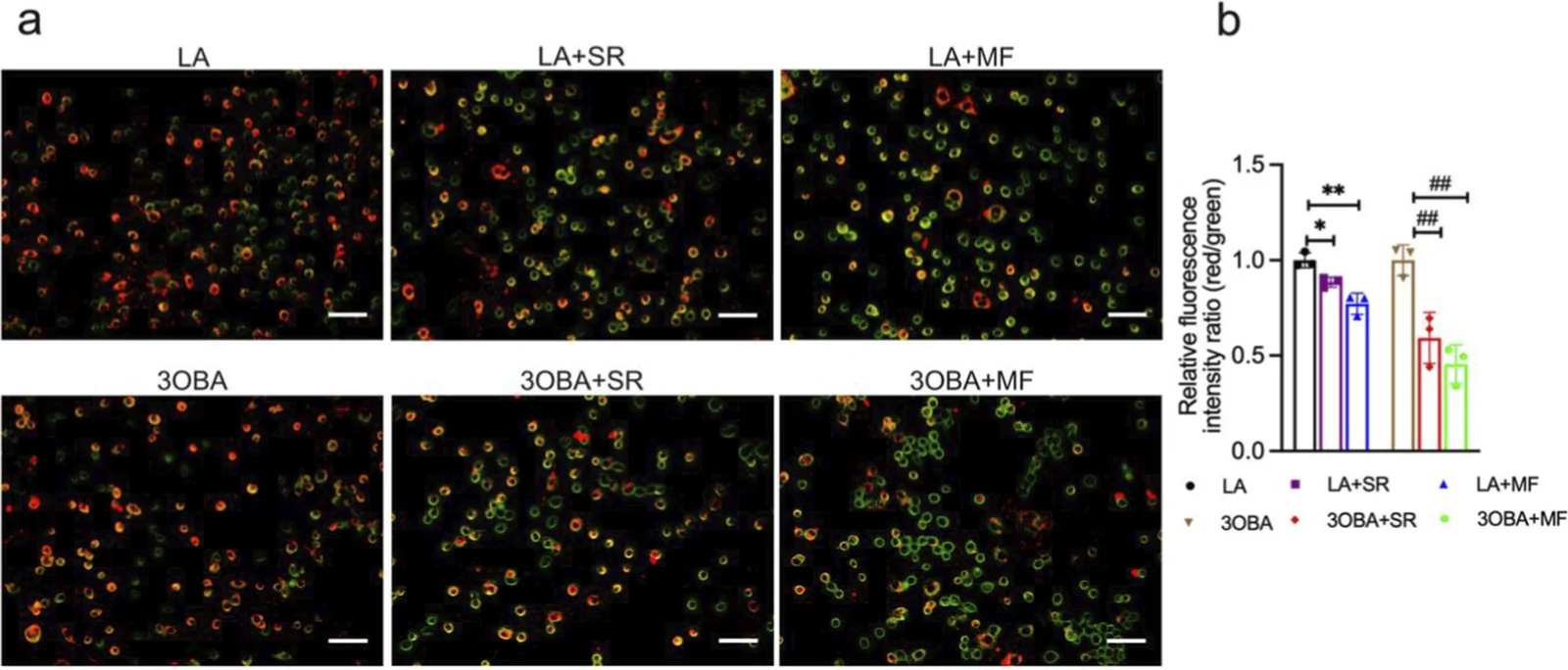
Effects of SR-18292 and MF18 on MMP in Neuro-2A cells. **(a)** Images show JC-1 staining representative of mitochondrial membrane potential (scale bar = 100 μm), where red and green fluorescence correspond to JC-1 aggregates and monomers, respectively. **(b)** Bar graphs depict the quantification of mitochondrial membrane potential, determined by the fluorescence intensity ratio (red/green) from three replicates. Data are expressed as mean ± SD. Statistical significance: *p < 0.05, **p < 0.01 versus the LA group. ##*p* < 0.01 versus the 3OBA group.

### SR-18292 and MF18 inhibit OXPHOS and ATP production in Neuro-2A cells

We next determined how impairing biogenesis or fusion affects mitochondrial energy output by measuring OXPHOS gene expression and ATP levels. SR-18292-mediated PGC-1α inhibition significantly downregulated key OXPHOS genes: *Uqcrc1* (complex III, Figure 9c), *COX5b* (complex IV, Figure 9d), and *Atp5a1* (ATP synthase, Figure 9e), accompanied by reduced cellular ATP levels (Figure 9f). A similar impairment was observed with the fusion inhibitor MF18, which decreased expression of *NDUFS8*, *SDHb*, *COX5b*, and *Atp5a1* (Figures 9a, b, d, e) and reduced ATP levels compared to lactate treatment alone (Figure 9f). Importantly, both inhibitors completely attenuated 3OBA-induced OXPHOS enhancement at the transcriptional level. Regarding ATP production, MF18 treatment showed a decreasing trend in 3OBA-treated cells, although this reduction did not reach statistical significance (Figure 9f). These results establish that functional mitochondrial biogenesis and fusion pathways are crucial for the lactate/GPR81 axis to enhance oxidative phosphorylation and cellular energy status.

**FIGURE 9:**
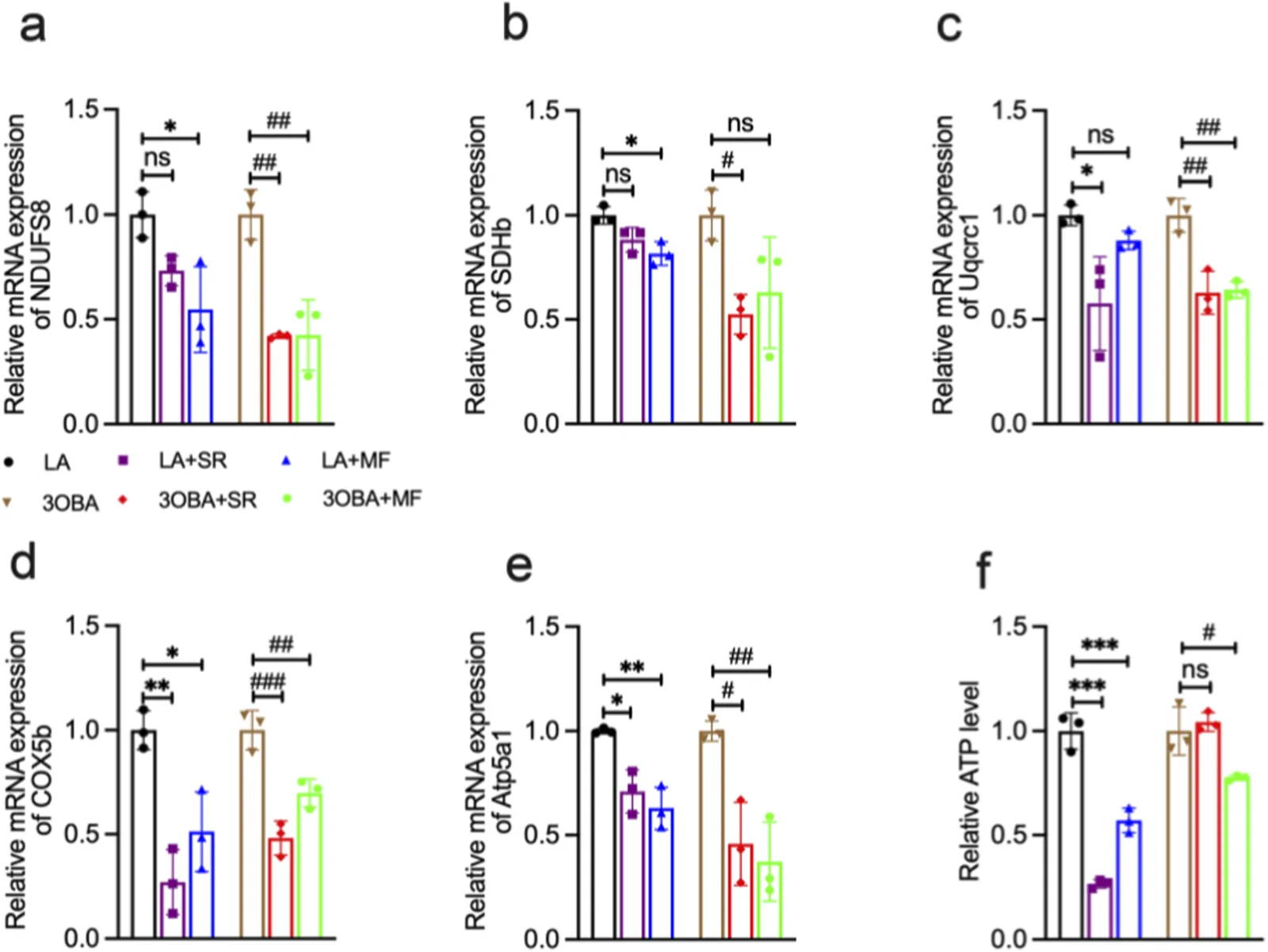
Effects of SR-18292 and MF18 on OXPHOS transcripts and ATP production in Neuro-2A cells. **(a-e)** Quantitative analysis (qRT-PCR) of *NDUFS8*, *SDHb*, *Uqcrc1*, *COX5b*, and *Atp5a1* transcript levels, derived from three independent replicates. **(f)** Quantification of relative ATP levels, derived from six independent replicates. Data are expressed as mean ± SD. Statistical significance: *p < 0.05, **p < 0.01, ***p < 0.001 versus the LA group. #*p* < 0.05, ##*p* < 0.01, ###*p* < 0.001 versus the 3OBA group. n. s., no significant difference.

### SR-18292 and MF18 impair the distribution of mitochondria within the neurite of Neuro-2A cells

Mitochondrial distribution was visualized in neurite of Neuro-2A cells via Mito-Tracker Red CMXRos staining. Both SR-18292 (LA + SR) and MF18 (LA + MF) treatment significantly decreased mitochondrial density within neurites compared to LA-treated cells (Figure 10a–c). This phenotype was similarly observed in 3OBA-treated cells following pharmacological inhibition (Figure 10d–f), confirming that both biogenesis and fusion are required for proper mitochondrial distribution in neuronal processes. The findings are consistent with a model wherein lactate/GPR81 signaling facilitates mitochondrial distribution in neurites by concurrently supporting biogenesis and fusion.

**FIGURE 10:**
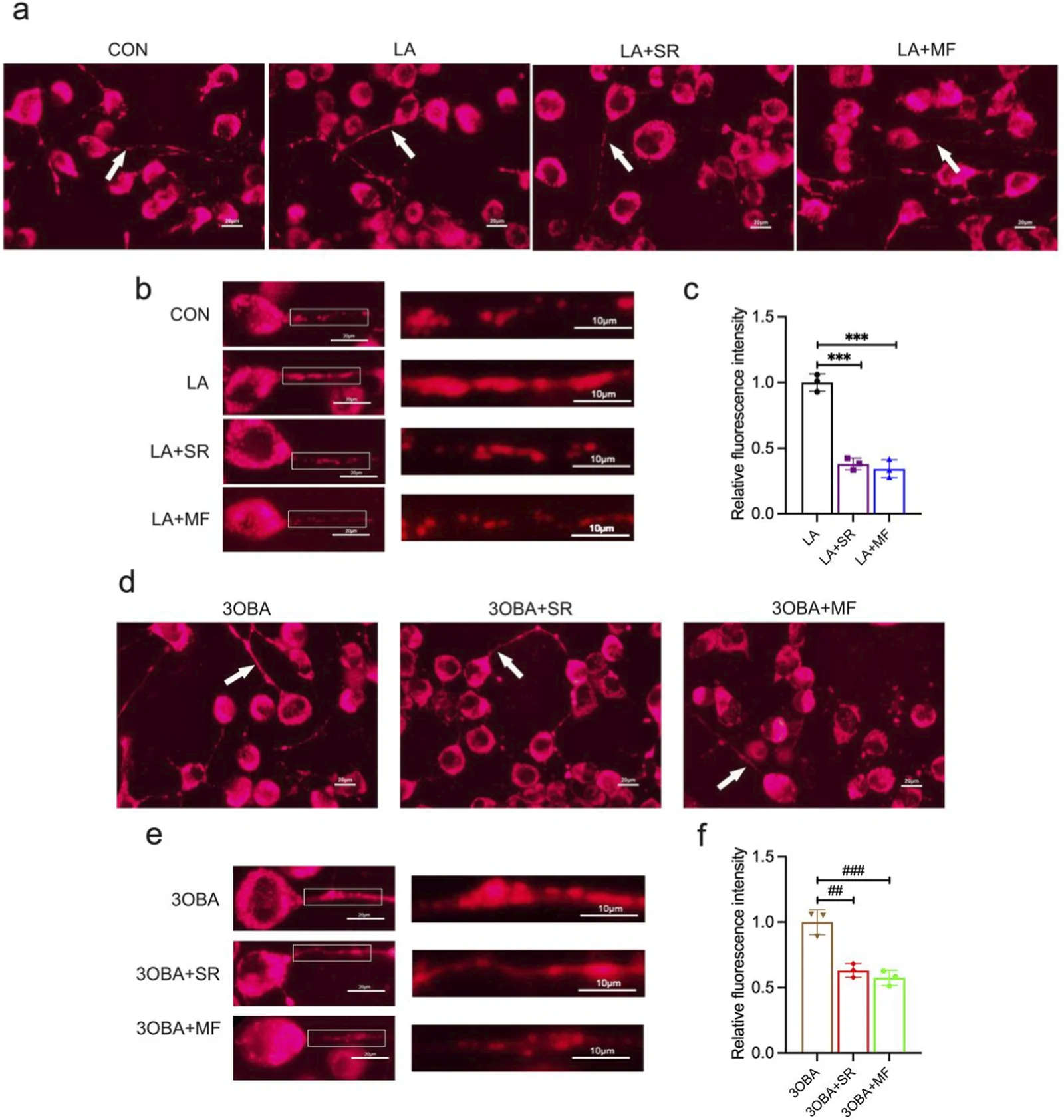
Effects of SR-18292 and MF18 on the distribution of mitochondria in neurite of Neuro-2A cells. **(a, d)** Full-field overview images of monolayer cells. **(b, e)** Higher magnification cropped images with rectangular boxes indicating the neurite regions of interest (ROIs) used for fluorescence quantification; the soma was excluded from analysis. **(c, f)** Bar graphs depict the quantification of fluorescence intensity, derived from three independent replicates. Data are expressed as mean ± SD. Statistical significance: ***p < 0.001 versus the LA group. ##*p* < 0.01, ###*p* < 0.001 versus the 3OBA group.

### Impairment of mitochondrial biogenesis or fusion downregulates synaptic plasticity-related protein expression in Neuro-2A cells

To further investigate whether the reduced mitochondrial distribution within the neurite of Neuro-2A cells impairs synaptic plasticity, we examined both gene and protein expression. In SR-18292-treated cells (LA + SR), we observed coordinated downregulation of key plasticity-related genes: *Arc* (Figure 11a), *Fos* (Figure 11b), *Bdnf* (Figure 11d), and *Syp* (Figure 11e), accompanied by reduced SYN (Figure 11h) and PSD95 (Figure 11i) protein expression. While MF18 treatment (LA + MF) primarily affected *Syp* gene expression (Figure 11e), it similarly diminished synaptic protein levels (Figures 11h,i). Notably, both inhibitors completely abolished 3OBA-induced upregulation of plasticity markers, including *Arc*, *Fos*, *Bdnf*, and *Syp* gene expression (Figures 11a,b,d,e) as well as BDNF, SYN and PSD95 proteins (Figures 11g–i). Regarding Egr1 (Figure 11c), both SR-18292 and MF18 treatment induced a decreasing trend in its mRNA expression, although these changes did not reach statistical significance. These results demonstrate that mitochondrial biogenesis and fusion are integral to maintaining the expression of synaptic gene and protein, and that lactate/GPR81 signaling enhances synaptic function through these mitochondrial pathways. Representative immunoblots are shown in Figure 11f.

**FIGURE 11:**
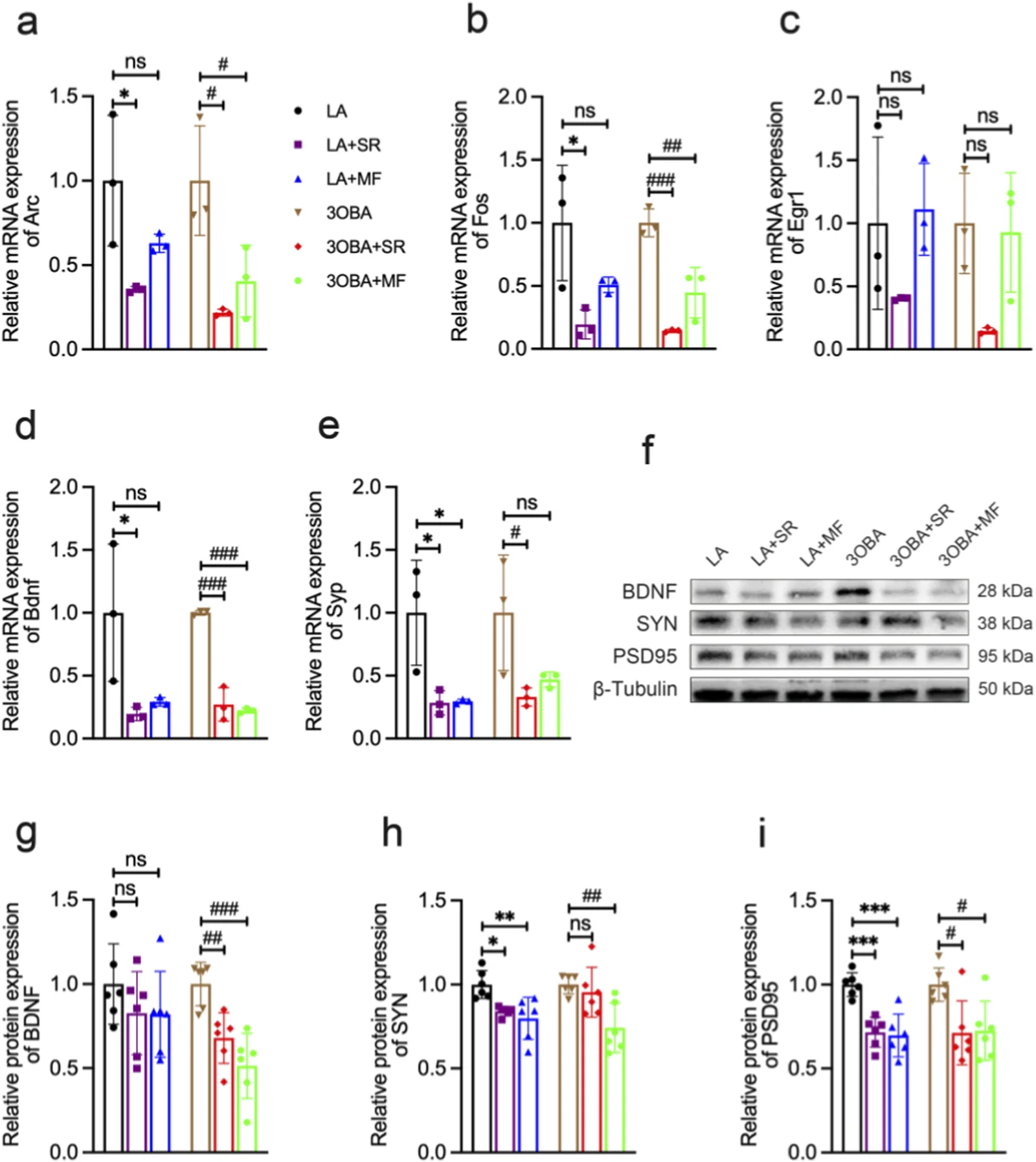
Effects of mitochondrial dysfunction on expression of genes and proteins related to synaptic plasticity in Neuro-2A cells. **(a-e)** Quantitative analysis (qRT-PCR) of *Arc*, *Fos*, *Egr1*, *bdnf*, *and Syp*, derived from three independent replicates. **(f)** Exemplary Western blot image. **(g-i)** Bar graphs depict the quantified protein levels for BDNF, SYN, and PSD95, derived from six independent replicates. Data are expressed as mean ± SD. Statistical significance: *p < 0.05, **p < 0.01, ***p < 0.001 versus the LA group. #*p* < 0.05, ##*p* < 0.01, ###*p* < 0.001 versus the 3OBA group. n. s., no significant difference.

## Discussion

Our study shows that L-lactate and a GPR81 agonist augmented mitochondrial biogenesis, fusion, ATP generation, and synaptic plasticity-related protein expression in primary hippocampal cells. In contrast, pharmacological impairment of these mitochondrial processes negated the synaptic benefits of lactate/GPR81 activation in Neuro-2A cells. Collectively, our findings provide the first evidence that lactate/GPR81 signaling enhances synaptic plasticity by promoting mitochondrial biogenesis and fusion. This mechanism establishes a functional link between lactate-mediated signaling, mitochondrial function, and synaptic plasticity.

Lactate is increasingly recognized as a key modulator of synaptic plasticity. For instance, impaired lactate transport reduces plasticity-related gene expression, arborization, and spine density, all rescued by L-lactate (Tan et al., 2022). Similarly, neonatal sevoflurane exposure-induced deficits in hippocampal neurogenesis and synaptic plasticity can be rescued by L-lactate treatment (Qiu et al., 2023). Supporting these findings, L-lactate preconditioning upregulates GPR81 and synaptic markers (PSD95, GAP43, BDNF) while improving neurological outcomes after traumatic brain injury in rats (Zhai et al., 2020). Our current results align with these observations, demonstrating that both L-lactate and a GPR81 agonist elevate synaptic plasticity-related protein expression in primary hippocampal cells. This aligns with our previous work showing reduced synaptic protein levels in GPR81-deficient mice (Shang et al., 2023). Together with these results, lactate may promote synaptic plasticity primarily through GPR81. Our findings, while revealing a neuron-intrinsic mechanism, do not capture the complexity of the neurovascular unit. In the brain, astrocytes are the primary source of lactate, and their interaction with neurons, as well as signals from microglia and endothelium, are critical for shaping synaptic plasticity. Therefore, the translational relevance of our monoculture findings awaits validation in more complex systems.

Maintaining synaptic energy homeostasis presents a unique challenge for neurons, where mitochondria play a central role by supplying ATP critical for synaptic transmission (Mattson et al., 2008; Subramanian and Jonas, 2021; Li et al., 2020). Emerging evidence positions lactate as a key modulator of mitochondrial function. In L6 cells, L-lactate upregulates PGC-1α expression and enhances NRF2 DNA-binding activity (Hashimoto et al., 2007), induces a protective ROS response in neuroblastoma cells (Tauffenberger et al., 2019), and increases mtDNA content alongside biogenesis markers (PGC-1α and TFAM) in podocytes (Audzeyenka et al., 2024). Our findings extend these observations to hippocampal cells, demonstrating L-lactate-induced upregulation of mitochondrial biogenesis markers (PGC-1α, NRF1/2, TFAM) - with TFAM serving as a critical coordinator of mtDNA transcription and nuclear-encoded OXPHOS genes (Dominy and Puigserver, 2013). Notably, we also observed enhanced expression of mitochondrial fusion proteins, consistent with their established roles in mtDNA stability (Yang et al., 2015) and energy delivery (Skulachev, 2001). Importantly, the concomitant increase in ATP production in our experiments provides direct evidence that lactate enhances mitochondrial respiratory function, thereby meeting the elevated energy demands of synaptic plasticity. Notably, systemic lactate administration in diabetic mice rescues mitochondrial and synaptic integrity, improving cognitive function (Wu et al., 2025). This underscores lactate’s central role in mitochondrial regulation and its translational potential for cognitive disorders.

An interesting observation in our study is the differential regulation of mitochondrial fusion proteins. While OPA1 and MFN2 were significantly upregulated by L-lactate, MFN1 protein levels were not significantly altered by L-lactate treatment, despite being significantly induced by the GPR81 agonist 3OBA. This differential response suggests that MFN1, MFN2, and OPA1 may be subject to distinct regulatory mechanisms downstream of lactate/GPR81 signaling. MFN1 and MFN2, though both core components of the mitochondrial fusion machinery, are functionally non-redundant; MFN2 plays a more prominent role in mitochondrial bioenergetics, axonal transport, and ER-mitochondria tethering, whereas MFN1 is primarily involved in outer membrane fusion (Chen et al., 2003; Detmer and Chan, 2007; Filadi et al., 2015). The preferential upregulation of MFN2 and OPA1 by L-lactate may reflect a specific adaptive response to support activity-dependent mitochondrial function in neurons, whereas MFN1 may be regulated through distinct pathways that are differentially engaged by exogenous L-lactate versus direct GPR81 agonism. Further investigation is needed to elucidate the molecular basis of this differential regulation. An interesting observation in our study is the discordance between MFN2 mRNA and protein levels following MF18 treatment. While MF18 significantly reduced MFN2 protein expression, its mRNA levels showed only a decreasing trend that did not reach statistical significance. This suggests that the inhibitory effect of MF18 on MFN2 may occur primarily at the post-transcriptional or post-translational level, rather than through transcriptional suppression. Several mechanisms could account for this discrepancy. First, MF18 may promote MFN2 protein degradation via the ubiquitin-proteasome system, as mitochondrial fusion proteins are known to undergo rapid turnover through proteasome-dependent pathways (Leboucher et al., 2012; Sugiura et al., 2013). Second, MF18 treatment may reduce MFN2 protein stability or impair its translation efficiency without affecting mRNA transcription. Third, it is also plausible that the modest downward trend in MFN2 mRNA, when combined with post-transcriptional mechanisms, collectively contributes to the significant reduction in protein levels. Future studies using cycloheximide chase assays or proteasome inhibition experiments will be informative to directly assess MFN2 protein stability and turnover under these conditions.

To investigate GPR81’s role in lactate-mediated mitochondrial enhancement, we examined the effects of a specific GPR81 agonist in primary hippocampal cells. Notably, the agonist recapitulated all observed effects of L-lactate, including elevated ATP levels and upregulation of mitochondrial biogenesis/fusion markers, indicating that GPR81 may be involved in lactate-mediated mitochondrial function promotion. This conclusion is further supported by accumulating evidence across diverse systems. For example, both L-lactate and GPR81 agonist increased protein expression associated with mitochondrial function in C2C12 cells (Sun et al., 2016). GPR81 agonist treatment enhanced ATP levels in retinal explants and promoted survival of retinal ganglion cells (Vohra et al., 2022). In human glioblastoma cell lines, both L-lactate and GPR81 agonist increased gene expression associated with mitochondrial function, including *PGC-1α*, *COX IV*, *COX II*, and *ATPsyn* (Longhitano et al., 2022b). Conversely, genetic studies demonstrate that GPR81 knockdown diminishes gene expression of PGC-1α and mitochondrial activity, which led to reduced cancer cell survival (Roland et al., 2014). Moreover, lactate treatment increased the ATP production in cancer cells, which was attenuated by GPR81 knockdown, suggesting that GPR81 regulated lactate-dependent ATP production (Ishihara et al., 2022). Together, these consistent findings across multiple models establish GPR81 activation as the principal mechanism through which lactate enhances mitochondrial function. We acknowledge that increased GPR81 protein expression alone does not demonstrate receptor activation. However, we observed that both L-lactate and 3OBA treatment significantly increased ERK1/2 phosphorylation in primary hippocampal cells, a well-established downstream signaling event of GPR81 activation (Shang et al., 2023) (Shang et al., 2023). This, together with the increased ATP production and enhanced mitochondrial and synaptic protein expression, collectively supports that GPR81 is functionally activated by lactate and 3OBA in our experimental system.

Our earlier work identified the GPR81-ERK1/2 pathway as mediating lactate’s effects on mitochondrial function in hippocampi of mice (Shang et al., 2023). This led us to a central hypothesis: that lactate/GPR81 promotes synaptic plasticity by optimizing mitochondrial activity. To mechanistically dissect this, we impaired mitochondrial biogenesis or fusion in Neuro-2A cells. Our results showed that both PGC-1α inhibitor and fusion inhibitor impaired mitochondrial function, including the opening of the mitochondrial permeability transition pore, mitochondrial membrane potential, OXPHOS, and ATP levels. In addition, mitochondrial biogenesis and fusion impairments contributed to a reduction in mitochondrial distribution within the neurites of Neuro-2A cells. Our MitoTracker Red CMXRos staining revealed that inhibiting either mitochondrial biogenesis (via SR-18292) or fusion (via MF18) significantly reduced mitochondrial distribution within neurites of Neuro-2A cells, suggesting that both processes are required for proper mitochondrial localization in neuronal processes. Notably, these findings reflect steady-state mitochondrial distribution in fixed cells rather than dynamic trafficking, as MitoTracker Red CMXRos primarily labels mitochondria in a membrane potential-dependent manner. Our quantitative analyses were performed on neurite ROIs with the soma excluded, and the results are supported by independent JC-1 MMP measurements showing consistent patterns of mitochondrial dysfunction. Live-cell imaging studies will be required to assess mitochondrial motility and dynamics in future investigations.

Collectively, these findings indicate that lactate/GPR81-regulated mitochondrial function are dependent upon mitochondrial biogenesis and fusion. Our evidence align with previous reports showing that PGC-1α knockdown reduced ATP levels while its overexpression elevated ATP levels (Cheng et al., 2012). Moreover, knockdown of MFN1 and OPA1 decreased mitochondrial respiration in old normal human fibroblasts (NHFs) (Son et al., 2017). Previous research has also found that dysfunction of the electron transport chain and loss of mtDNA were found in Purkinje Cells Lacking MFN2 (Chen et al., 2007). Additionally, MFN2 knockdown had significantly impaired OXPHOS, mtDNA nucleoids, and electron transport activity in neurons (Motori et al., 2020). Importantly, our study revealed that disrupting mitochondrial biogenesis and fusion correspondingly downregulated synaptic plasticity-related protein expression, establishing a direct mechanistic link between lactate/GPR81-enhanced mitochondrial function and synaptic plasticity. This is further corroborated by studies in cultured hippocampal cells, where PGC-1α overexpression boosts dendritic spine density and synaptic protein levels, whereas its deficiency produces the opposite effect, reducing spines and synapse number (Cheng et al., 2012). These findings underscore a critical role for PGC-1α in the formation and maintenance of hippocampal synapses. In addition, MFN2 KO induced neurite degeneration in neurons and neuronal death in mice. In contrast, MFN2 overexpression rescued mitochondrial morphology, thereby preventing neurite degeneration and rescuing neuronal death (Motori et al., 2020). Together, these results demonstrate that mitochondrial biogenesis and fusion serve as essential downstream effectors of lactate/GPR81 signaling in promoting synaptic protein expression.

Our findings, while revealing a neuron-intrinsic mechanism, do not capture the complexity of the neurovascular unit. In the brain, astrocytes are the primary source of lactate, and their interaction with neurons, as well as signals from microglia and endothelium, are critical for shaping synaptic plasticity. Therefore, the translational relevance of our monoculture findings awaits validation in more complex systems. Additionally, while our primary hippocampal neuron data demonstrate that L-lactate and GPR81 activation enhance mitochondrial and synaptic protein expression in a physiologically relevant neuronal system, the mechanistic inhibitor experiments (SR-18292 and MF18) were performed exclusively in Neuro-2A cells. This cell line, while widely used as a neuronal model, is a proliferative neuroblastoma line and its transformed nature limits its ability to fully recapitulate the complex biology of post-mitotic neurons. Therefore, our findings regarding the mechanistic dependency on PGC-1α and mitochondrial fusion should be interpreted as supporting a mechanistic model that awaits further validation in primary neurons and *in vivo*. We acknowledge this as a key limitation of the present study. Furthermore, we acknowledge that our conclusions regarding synaptic plasticity are based exclusively on molecular readouts (protein and mRNA expression of synaptic markers), which, while well-established correlates of synaptic function, do not constitute direct evidence of functional synaptic plasticity. Electrophysiological recordings (e.g., LTP or mEPSC measurements) and morphological analyses (e.g., dendritic spine density) will be required to confirm the functional significance of our findings.

In summary, our study demonstrates that lactate promotes mitochondrial biogenesis and fusion via GPR81 activation, leading to enhanced expression of synaptic plasticity-related proteins in hippocampal cells. While our *in vitro* findings have elucidated key cellular mechanisms and demonstrated that these effects are dependent on PGC-1α-mediated biogenesis and mitochondrial fusion, we acknowledge that the functional implications of these molecular changes for synaptic transmission and plasticity remain to be established. Future investigations using electrophysiological recordings and morphological analyses, together with *in vivo* loss-of-function models, will be essential to confirm the physiological relevance of this signaling pathway. Given the established link between mitochondrial dysfunction and cognitive impairment, our findings suggest that the lactate-GPR81-mitochondria axis may represent a potential target for maintaining synaptic integrity, and this possibility requires direct functional validation in future studies.

## Conclusion

In summary, we demonstrate that L-lactate and GPR81 activation enhance mitochondrial biogenesis, fusion, ATP production, and synaptic plasticity-related protein expression in primary hippocampal cells. Mechanistic studies in Neuro-2A cells indicate that these effects are dependent on PGC-1α-mediated mitochondrial biogenesis and mitochondrial fusion. While these findings support a model in which lactate/GPR81 signaling promotes synaptic function through mitochondrial enhancement, future studies in primary neurons and *in vivo* are warranted to confirm the physiological relevance of this mechanism.

## Data Availability

The original contributions presented in the study are included in the article/supplementary material; further inquiries can be directed to the corresponding author.
